# The Laboratory and Semi-Field Larvicidal Effects of Essential Oil Extracted from *Feronia limonia* against *Anopheles arabiensis* Patton

**DOI:** 10.1155/2023/5907603

**Published:** 2023-02-22

**Authors:** Eliningaya J. Kweka, France P. Mdoe, Norah N. Lowassari, Venugopalan Venkatesalu, Annadurai Senthilkumar

**Affiliations:** ^1^Department of Medical Parasitology and Entomology, School of Medicine, Catholic University of Health Sciences, P.O. Box 1464 Mwanza, Tanzania; ^2^Tropical Pesticides Research Institute, P.O. Box 3024 Arusha, Tanzania; ^3^Marian University College, P.O. Box 47, Bagamoyo, Pwani, Tanzania; ^4^Department of Botany, Annamalai University, Annamalai Nagar, Tamil Nadu 608 002, India

## Abstract

This study intended to evaluate the larvicidal activity of *Feronia limonia* leaf essential oil against the wild population of *Anopheles arabiensis* Patton larvae in laboratory and semi-field environments. Larvae mortality was observed after 12, 24, 48, and 72 hours of exposure. In laboratory condition, the essential oil showed good larvicidal activity against *An. arabiensis* (LC_50_ = 85.61 and LC_95_ = 138.03 ppm (after 12 hours); LC_50_ = 65.53 and LC_95_ = 117.95 ppm (after 24 hours); LC_50_ = 32.18 and LC_95_ = 84.59 ppm (after 48 hours); LC_50_ = 8.03 and LC_95_ = 60.45 ppm (after 72 hours), while in semi-field experiments, larvicidal activity was (LC_50_ = 91.89 and LC_95_ = 134.93 ppm (after 12 hours); LC_50_ = 83.34 and LC_95_ = 109.81 ppm (after 24 hours); LC_50_ = 66.78 and LC_95_ = 109.81 (after 28 hours); LC_50_ = 47.64 and 90.67 ppm (after 72 hours). These results give an insight on the future use of *F. limonia* essential oils for mosquitoes control.

## 1. Introduction

Mosquito control in recent past has been challenged by the wide spread of insecticide resistance among the vector populations [1, 2]. The decline of efficacy of currently used vector control tools (long-lasting insecticidal nets and indoor residual spray) have threatened the achieved efforts across Sub-Saharan Africa [1, 2]. The search for alternative insecticides with different mode of action is of paramount importance. The targeting of breeding habitats has shown a great impact on larvae and subsequently reduction of adult mosquitoes and disease incidences [3]. The search of the natural products to overcome the resistance by targeting the aquatic stages of mosquitoes has shown an effect in laboratory and semi-field evaluation [3]. The use of botanical extracts for pests control has shown to have no side effect on environment and non-targeted organisms [4, 5]. Previous studies have shown that the essential oil from plants displayed a great impact on larvicidal, adulticidal, and repellent effects [6]. To date, the plant extracts resistance by vectors has not been reported [6]. The aim of this study was to assess the larvicidal activities of *Feronia limonia* leaf essential oil against *An. arabiensis*.

## 2. Material and Methods

### 2.1. Plant Collection and Essential Oil Extraction

The leaves of *F. limonia* were collected from Keerapalayam [11°26′;03 N, 079°39′;02 E], Cuddalore District, Tamil Nadu, India, and the voucher specimen (AUBOT# 209) is deposited at the Herbarium, Department of Botany, AU. The fresh leaves were subjected to hydro-distillation using Clevenger-type apparatus for 4 hours. The essential oil was dried over anhydrous sodium sulphate, and the purified essential oil was stored at +4°C for mosquito larvicidal activity.

The essential oil volatile constituents were determined by Gas chromatography–mass spectrometry (GC-MS) by using Varian 3800 Gas Chromatography equipped with Varian 1200 L single quadrupole mass spectrometer. The mass spectrometer was operated in the electron impact (IE) mode at 70 eV. Ion source and transfer line temperature were kept at 250°C. The mass spectra were obtained by centroid scan of the mass range from 40 to 1000 amu. The compounds were identified based on the comparison of their retention indices (RI), retention time (RT), and data bank mass spectra of Wiley library (Table 1).

### 2.2. Mosquito Larvae Rearing

The *An. arabiensis* Patton eggs were obtained from wild gravid mosquitoes collected from cowsheds in Lower Moshi near rice irrigation schemes. The wild population of *Anopheles gambiae* s.l. in this area has been confirmed to be composed of 100% *An. arabiensis* [7]. The larvae room was maintained at the temperature of 27 ± 2°C and relative humidity of 78 ± 2%. The larvae were fed with 0.003 g of Tetra mine per larva as shown in previous study [8]. The *An. arabiensis* larvae were reared until when they were stage three instars and used for screening as per WHO guidelines.

### 2.3. Mosquito Larvicidal Assay

The *F. limonia* essential oil was dissolved in 1 ml of acetone. Then, serial dilution was made from this stoke solution into six concentrations 3.125, 6.25, 12.5, 25, 50, and 100 ppm with distilled water as per WHO guidelines. Each concentration had six replicates, each with twenty-third instar larvae of *An. arabiensis*. There were two controls: one control (C1) contained distilled water, while the other (C2) contained 1 ml aqueous solution of acetone. During these assays, food was provided to the larvae after 24 hours. The mortality of larvae was monitored after 12, 24, 48, and 72 hours of exposure period in both treatments and controls. The dead and moribund larvae were both considered dead. Experiments were set in both laboratory and semi-field conditions as described elsewhere [9].

### 2.4. Data Analysis

Data were entered into excel sheet and transferred into IBM SPSS Version 26 (IMB Corp., Armonk, NY, USA) for analysis. The lethal concentrations LC_50_ and LC_95_ and their 95% confidence limit of upper and lower confidence levels were calculated by probit analysis [10]. The comparison of larvae mortality among treatments, between treatments and control, and between laboratory and semi-field environment were conducted using Chi-square test.

## 3. Results

### 3.1. Chemical Composition

The GC-MS chemical analysis of the *F. limonia* leaf essential oil revealed 51 chemical compounds with estragole as the highest abundant compound with 34.69%, while *cis*-dihydro-*β*-terpineol and *ρ*-Cymene were revealed to have the least amount of 0.03% each. The other 48 compounds occurred in different percentage compositions (Table 1).

### 3.2. Mortality Effect of the Essential Oils

**Figure 1:**
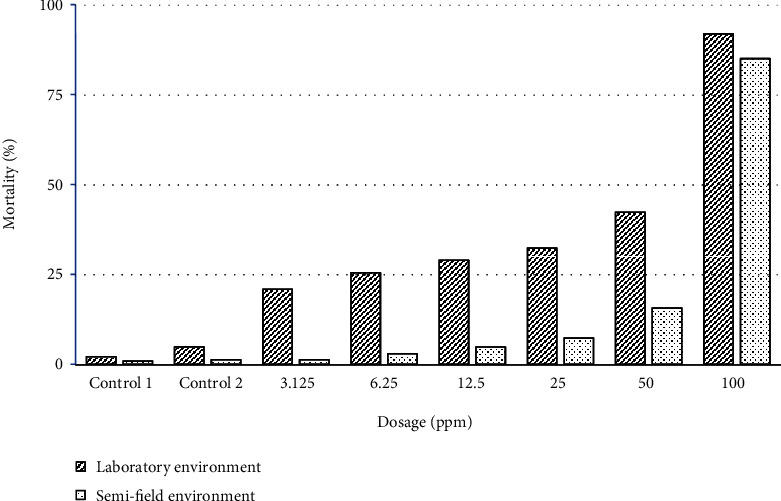
Effect of dosage on mortality of *An. arabiensis* third instar larvae under both laboratory and semi-field environment. Controls: ∗C1—water and C2—aqueous solution of 1 ml acetone.

The mortality of the larvae of *An. arabiensis* from 12 to 72 hours of observation in both laboratory and semi-field environment was found to be dosage dependent (Figure 1), and the proportion of larvae that died between laboratory and semi-field environments was found to have statistically significant difference (Table 2). The observed mortality rate was higher in the laboratory environment as compared to semi-field environment, it ranged between 20.83% in 3.125 ppm and 91.88% in 100 ppm (Table 2). Also, the mortality effect was found to be exposure time dependent, with percentage mortality significant different revealed in some time intervals (Table 3).

### 3.3. Lethal Dose

The lethal dose enough to kill 50% (LC_50_) and 95% (LC_95_) of the larvae exposed varied with exposure time in both laboratory and semi-field environments (Table 4). In both LC_50_ and LC_95_, the lowest values were found in laboratory than in the semi-field conditions (Table 4). The proportions of lethal dose comparison were found to differ significantly between laboratory and semi-field experiments (Table 4).

## 4. Discussion

The findings of this study have highlighted the impact of *F. limonia* essential oil against *An. arabiensis* larvae. The mortality of the larvae in both laboratory and semi-field was found to be dosage dependent with higher mortality observed in laboratory. These findings are similar to the previous study conducted using larvae of *Aedes aegypti, Anopheles stephensi*, *Culex quinquefasciatus*, and *An. gambiae s.s.* having more mortality in laboratory than semi-field [11]. This mortality decreases in semi-field compared to the laboratory and it might have been attributed to the exposure of the essential oil to the sunlight, which might cause compound degradation into secondary metabolites with low toxicity [12]. To maintain the effectiveness of the botanical larvicides, repeated application is needed frequently [13]. The modification of the plant-based biolarvicides is of paramount importance to extend its longevity in environment by making it in slow-release technology. This technology has been practical to *Bacillus thuringiensis israelensis* (Bti), which in application lasts for 5–7 days [14, 15], but with improved slow-release technology, it has lasted active against larvae for 6 months with no effects to non-targeted organisms [3, 16].

In recent years, the non-particle technologies have enhanced natural products to be more stable and effective. The recent nanoparticles incorporated in natural products have seen to be effective for public health and agriculture pests [17].

The larval source management currently is mostly done with frequent application of organophosphate [15] and insect growth regulators [18, 19]. So, taking up plant- and fungal-based natural product in improved synthesis in small-scale trials can be paving a way to manage insecticide resistance and reduce vector abundance. The findings of this study have shown that the essential oils of *F. limonia* have impact on mosquito mortality, but in semi-field environment has to be modified to maintain the laboratory observed mortality results.

The larvicidal mortality seen in this study might have been caused by the occurrence of *β*-pinene and estragole (methyl chavicol). Estragole is a common chemical constituent in plants' essential oils [20]. Essential oils with methyl chavicol from different have shown high mortality against mosquito larvae. Also, this compound has shown to exhibit fumigant and contact toxicity against *Ceratitis capitata*, *Bactrocera dorsalis*, *Bactrocera cucurbitae* [21], and other storage post-harvest pests [22, 23]. In *F. Limonia*, *β*-pinene occurrence was higher and past studies have shown high mortality effect of larvae [24]. The *β*-pinene-rich in essential oil has shown to impact mortality of Aedes *aegypti* fourth instar *Ae. aegypti* larvae. Also, there might be an impact of elements considered to be minor components of the essential oils in larvicidal activity of essential oils [25]. In *Ocimum suave* essential oils, the linalool among the least occurring ingredient was found to be a major source of mortality [26]. In this case, the natural products from plants have shown to be the major alternative of synthetic pesticides if well moderated and composed.

## 5. Conclusion

The findings of this study have shown that the essential oils of *F. limonia* have impact on mosquito mortality, but in semi-field environment has to be modified to maintain the laboratory observed mortality results.

## Figures and Tables

**Table 1:** Chemical compounds of the essential oil of *F. limonia* leaves.

No.	Retention time (min)	Retention indices (RI)	Chemical compounds	Composition (%)
1	3.996	801	Hexanal	0.07
2	5.226	924	*α*-Thujene	0.04
3	5.373	932	*α*-Pinene	1.75
4	5.716	946	Camphene	0.14
5	6.172	969	Sabinene	2.41
6	6.267	974	*β*-Pinene	23.59
7	6.486	988	Myrcene	0.17
8	6.829	1,004	(3*E*)-3-hexenyl acetate	0.10
9	7.007	1,014	*α*-Terpipene	0.10
10	7.168	1,020	*ρ*-Cymene	0.03
11	7.236	1,024	Limonene	2.27
12	7.271	1,025	*β*-Phellandrene	0.55
13	7.354	1,032	*β*-(*Z*)-ocimene	0.07
14	7.541	1,044	*β*-(*E*)-ocimene	0.90
15	7.764	1,054	*γ*-Terpinene	0.23
16	8.015	1,078	Camphenilone	0.08
17	8.240	1,084	Terpinolene	0.11
18	8.502	1,095	Linalool	3.97
19	8.948	1,119	Myrcenol	0.04
20	9.170	1,128	Allo-Ocimene	0.05
21	9.250	1,138	Geijerene	0.05
22	9.756	1,156	*cis*-Dihydro-*β*-terpineol	0.03
23	9.862	1,165	Borneol	0.79
24	10.156	1,195	Estragole	34.69
25	10.549	1,199	*γ*-Terpineol	0.09
26	11.478	1,239	*ο*-Anisaldehyde	0.12
27	11.54	1,249	(*Z*)-anethole	0.07
28	11.941	1,271	Citronellyl formate	0.12
29	12.378	1,274	Pregeijerene B	0.07
30	12.645	1,361	(*Z*)-*β*-damascenone	0.11
31	12.726	1,379	Geranyl acetate	0.08
32	12.83	1,389	*β*-Elemene	0.04
33	12.946	1,392	(*Z*)-jasmone	0.30
34	13.005	1,403	Methyl eugenol	6.50
35	13.275	1,408	(*Z*)-Caryophyllene	11.05
36	13.744	1,484	Germacrene D	1.05
37	14.091	1,498	*α*-Selinene	0.33
38	14.262	1,505	*α*-(*E*,*E*)-farnesene	0.55
39	14.8	1,548	Elemol	1.77
40	14.853	1,555	Elemicin	0.24
41	14.977	1,565	(3*Z*)-hexenyl benzoate	0.05
42	15.179	1,569	*γ*-Undecalactone	0.42
43	15.283	1,576	Santalenone	0.36
44	15.359	1,582	Caryophyllene oxide	0.47
45	15.693	1,608	Humulene epoxide II	0.04
46	15.878	1,632	(3*Z*)-Hexenyl phenyl acetate	0.05
47	15.967	1,645	Cubenol	0.10
48	16.009	1,652	*α*-Eudesmol	0.28
49	16.161	1,678	(*Z*)-Methyl epi-jasmonate	0.18
50	16.379	1,713	Longifolol	0.06
51	20.705	1,942	Phytol	3.27
Total				100

**Table 2:** Mortality by dosage in both laboratory and semi-field environment for *An. arabiensis* larvae.

Dosage (ppm)	Laboratory	Semi-field	*X* ^2^ (*p-*value)
	% mortality (95% CI)	% mortality (95% CI)	
C_1_	0.96 (0.24–1.68)	0.78 (0.16–1.40)	1.0 (1.000)

C_2_	4.83 (3.18–6.47)	1.25 (0.32–2.81)	1.0 (1.000)
3.125	20.83 (10.13–31.54)	1.25 (0.13–2.37)	17.75 (0.001)
6.25	25.42 (13.59–37.24)	2.92 (1.06–4.78)	23.43 (0.001)
12.5	28.96 (16.24–41.67)	4.79 (2.04–7.54)	28.11 (0.001)
25	32.29 (19.08–45.5)	7.29 (3.51–11.08)	19.91 (0.001)
50	42.29 (29.38–55.2)	15.63 (7.38–23.87)	16.42 (0.001)
100	91.88 (88.84–94.91)	85.0 (80.51–89.49)	2.41 (0.1208)

**Table 3:** Mortality effect over time in both laboratory and semi-field environment for *An. arabiensis* larvae.

Time (hours)	Lab	Semi-field	*X* ^2^ (*p*-value)
	% mortality (95% CI)	% mortality (95% CI)	
12	12.56 (3.72–21.4)	10.12 (2.26–17.98)	0.44 (0.506)
24	19.42 (9.96–28.88)	12.5 (3.34–21.66)	1.34 (0.247)
48	41.28 (32.9–49.66)	17.5 (7.86–27.14)	12.72 (0. 001)
72	63.41 (54.38–72.44)	27.38 (17.6–37.16)	26.18 (0.001)

**Table 4:** Mean lethal dose responses for *Anopheles arabiensis* larvae.

Time (hours)	LC	Laboratory	Semi-field
12	LC_50_	85.61 (77.89–93.80)	91.89 (86.86–97.01)
	LC_95_	138.03 (127.98–149.66)	134.93 (129.01–141.321)
24	LC_50_	65.53 (59.19–72.39)	83.34 (78.59–88.21)
	LC_95_	117.95 (108.72–128.82)	126.38 (120.62–132.63)
48	LC_50_	32.18 (27.56–37.04)	66.78 (62.75–70.98)
	LC_95_	84.59 (77.22–93.33)	109.81 (104.36–115.82)
72	LC_50_	0.03 (3.48–12.46)	47.64 (44.37–51.08)
	LC_95_	60.45 (54.37–67.52)	90.67 (85.76–96.15)

## Data Availability

All data generated in this study have been provided in the manuscript.
